# NLRP6 Inflammasome Modulates Disease Progression in a Chronic-Plus-Binge Mouse Model of Alcoholic Liver Disease

**DOI:** 10.3390/cells11020182

**Published:** 2022-01-06

**Authors:** Rebecca Elena Mainz, Stefanie Albers, Madhuri Haque, Roland Sonntag, Nicole Simone Treichel, Thomas Clavel, Eicke Latz, Kai Markus Schneider, Christian Trautwein, Tobias Otto

**Affiliations:** 1 Department of Internal Medicine III, University Hospital RWTH Aachen, 52074 Aachen, Germany; rebecca.mainz@rwth-aachen.de (R.E.M.); stalbers@ukaachen.de (S.A.); mhaque@ukaachen.de (M.H.); rsonntag@ukaachen.de (R.S.); kmschneider@ukaachen.de (K.M.S.); 2 Functional Microbiome Research Group, University Hospital RWTH Aachen, 52074 Aachen, Germany; ntreichel@ukaachen.de (N.S.T.); tclavel@ukaachen.de (T.C.); 3 Institute of Innate Immunity, University Hospital Bonn, University of Bonn, 53127 Bonn, Germany; eicke.latz@uni-bonn.de; 4 Department of Microbiology, Perelman School of Medicine, University of Pennsylvania, Philadelphia, PA 19104, USA; 5 Institute for Immunology, Perelman School of Medicine, University of Pennsylvania, Philadelphia, PA 19104, USA; 6 Institute for Diabetes, Obesity, and Metabolism, Perelman School of Medicine, University of Pennsylvania, Philadelphia, PA 19104, USA

**Keywords:** NLRP6, inflammasome, alcoholic liver disease, ALD, gut microbiota, intestinal microbiota, gut-liver axis

## Abstract

A considerable percentage of the population is affected by alcoholic liver disease (ALD). It is characterized by inflammatory signals from the liver and other organs, such as the intestine. The NLR family pyrin domain containing 6 (NLRP6) inflammasome complex is one of the most important inflammatory mediators. The aim of this study was to evaluate a novel mouse model for ALD characterized by 8-week chronic-plus-binge ethanol administration and to investigate the role of NLRP6 inflammasome for intestinal homeostasis and ALD progression using *Nlrp6^-/-^* mice. We showed that chronic-plus-binge ethanol administration triggers hepatic steatosis, injury, and neutrophil infiltration. Furthermore, we discovered significant changes of intestinal microbial communities, including increased relative abundances of bacteria within the phyla *Bacteroidota* and *Campilobacterota*, as well as reduced *Firmicutes*. In this ALD model, inhibiting NLRP6 signaling had no effect on liver steatosis or damage, but had a minor impact on intestinal homeostasis via affecting intestinal epithelium function and gut microbiota. Surprisingly, *Nlrp6* loss resulted in significantly decreased hepatic immune cell infiltration. As a result, our novel mouse model encompasses several aspects of human ALD, such as intestinal dysbiosis. Interfering with NLRP6 inflammasome activity reduced hepatic immune cell recruitment, indicating a disease-aggravating role of NLRP6 during ALD.

## 1. Introduction

Alcoholic liver disease (ALD) encompasses a spectrum of diseases ranging from simple liver steatosis and alcoholic hepatitis to liver cirrhosis and hepatocellular carcinoma. In Europe, about 20 to 30% of the population are estimated to consume an excessive amount of alcohol [1]. About 90% of individuals with excessive alcohol consumption will develop liver steatosis, which will progress to steatohepatitis and even liver cirrhosis in about 50% and 25% of cases, respectively [2]. Although the determinants of disease progression in ALD are incompletely understood, the innate immune system and inflammatory signals between the intestine and the liver, termed the gut-liver axis, seem to play an important role [3].

In mouse models of ALD, chronic feeding of alcohol led to intestinal bacterial overgrowth, as well as intestinal dysbiosis, characterized by changes in the taxonomic composition of the intestinal microbiota [4]. In particular, alcohol feeding led to an increased presence of *Actinobacteria* and *Proteobacteria* while reducing *Bacteroidetes* and *Firmicutes* [5]. Furthermore, enhanced bacterial translocation can occur as a result of reduced expression of proteins involved in tight junctions between enterocytes [6]. Intestinal dysbiosis, bacterial overgrowth, and enhanced translocation of bacterial components from gut to liver may in turn impact liver function. Indeed, animal models of ALD have shown that repetitive exposure to bacterial LPS triggers activation of hepatic macrophages [7].

Gut-derived bacterial LPS is considered a pathogen-associated molecular pattern (PAMP) that is recognized by TLR4 receptors on hepatic macrophages, other immune cells, and liver parenchymal cells [7]. It likely acts as a first signal to activate inflammasome complexes during ALD by inducing expression of pro-interleukin 1 beta (IL1B) [8]. This includes the NLRP6 inflammasome, which contains NLR family pyrin domain containing 6 (NLRP6) as sensor molecule. Upon stimulation by damage-associated molecular patterns (DAMPs) as a second signal, inflammasome assembly triggers activation of Caspase-1, IL1B, interleukin 18 (IL18), and gasdermin D (GSDMD) [9]. While inflammasome activity is involved in many inflammatory processes including alcoholic hepatitis [10,11], the NLRP6 inflammasome has been implicated particularly in maintaining microbiome homeostasis in the gut by activating synthesis of anti-microbial peptides that control microbiota composition [12]. Interestingly, enhancing NLRP6 activity by virus-mediated overexpression of NLRP6 was shown to reduce liver steatosis, inflammation, and injury in alcohol-fed mice, suggesting a protective role of the NLRP6 inflammasome during ALD [13].

While many previous studies relied on chronic feeding of rodents with an ethanol-containing liquid diet (Lieber-DeCarli diet) [14,15], this well-known model of chronic alcohol abuse does not faithfully recapitulate the situation in human patients. Instead, chronic alcoholism in humans is often characterized by a drinking pattern that combines long-term alcohol consumption with episodes of very heavy drinking (episodic binge drinking). To better reproduce human drinking behavior, the lab of Bin Gao developed a chronic-plus-binge mouse model [16,17,18]. Although this “NIAAA model” combines 10-day ad libitum ethanol feeding (Lieber-DeCarli diet) with a single ethanol binge, it does not truly recapitulate the long-term characteristic of human alcoholism.

To determine the impact of chronic alcohol abuse and NLRP6 inflammasome activity on liver disease and intestinal homeostasis, we employed a novel long-term variation of this chronic-plus-binge “NIAAA model” of alcohol consumption in mice [18].

## 2. Materials and Methods

### 2.1. Mouse Generation and Housing

*Nlrp6* constitutive knockout (*Nlrp6*^-/-^) mice on a C57BL/6J genetic background were obtained from Eicke Latz (University Hospital Bonn, Bonn, Germany). Mouse lines for wild-type (WT) and *Nlrp6*^-/-^ mice were created from an initial heterozygous *Nlrp6*^+/−^ breeding pair. Homozygous offspring of this line were used to create individual WT and *Nlrp6*^-/-^ breedings, which were kept separate for at least two generations. These lines were kept in individually ventilated cages in the same room of the animal facility of the RWTH Aachen University. To avoid transmission of *Nlrp6*^-/-^ microbiome to WT mice (and vice versa), we did not allow any exchange of mice, cage, or nesting material between these two lines. All animals received humane care according to the criteria outlined in the “Guide for the Care and Use of Laboratory Animals” (8th edition, 2011), prepared by the National Academy of Sciences [19]. All mice used in these studies were maintained on 12-h dark/light cycles. All animal experiments were approved by the appropriate German authorities for Animal Welfare (LANUV, Recklinghausen, North Rhine-Westphalia, Germany; File Ref. 84-02.04.2015.A392).

### 2.2. Mouse Treatment

Ethanol treatment of male mice starting at the age of 10–14 weeks was performed by feeding mice ad libitum with the Lieber-DeCarli ethanol liquid diet (#F1258SP, Bioserv, Flemington, NJ, USA) containing 5% ethanol and maltose dextrin (#3585, Bioserv) over a period of eight weeks, as previously published [14,15]. To adapt mice to this diet, we added four “adaptation” days at the beginning with increasing concentrations of ethanol (i.e., 1%, 2%, 3%, and 4%). In addition, mice received ethanol binges using oral gavage after each full week of ethanol diet (i.e., a total of eight ethanol binges), with increasing doses of ethanol (i.e., three times with 3 g ethanol per kg body weight, then three times with 4 g/kg, then two times with 5 g/kg), similar to the previously published NIAAA model [18]. Mice were euthanized and analyzed five hours after the last ethanol binge administration. The overall survival rate of this treatment schedule was 75%. More specifically, seven out of ten WT mice (a survival rate of 70%) and five out of six Nlrp6^−/−^ mice (a survival rate of 86%) survived this treatment schedule.

Control treatment of male mice starting at the age of 10–14 weeks was performed by feeding mice ad libitum with the Lieber-DeCarli control liquid diet (#F1259SP, Bioserv) containing no ethanol over a period of eight weeks (plus four “adaptation” days), as previously published [14,15]. In addition, mice received control binges using oral gavage after each full week of control diet (i.e., a total of eight control binges), containing 9 g maltose dextrin (#3585, Bioserv) per kg of body weight, similar to the previously published NIAAA model [18]. Mice were euthanized and analyzed five hours after the last control binge administration. The overall survival rate of this treatment schedule was 100%.

Further analysis was performed with all mice that survived the complete treatment schedules, i.e., five mice for control WT, five mice for control Nlrp6^−/−^, seven mice for ethanol WT, and five mice for ethanol Nlrp6^−/−^.

### 2.3. Blood Analysis

Blood was collected from the vena cava inferior immediately after euthanizing mice. In the serum, ALT, AST, and GLDH activity (UV test at 37 °C), as well as blood alcohol concentration and triglyceride concentration, were measured at the Central Laboratory Facility of the University Hospital RWTH Aachen according to standard procedures.

### 2.4. Triglyceride Assay

For determination of intrahepatic triglyceride concentration, small pieces of livers (approx. 20 mg) were homogenized in a specific homogenization buffer (10 mM Tris, 2 mM EDTA, 0.25 M sucrose, pH 7.5) at 4 °C. Triglyceride concentration in the homogenate was measured using a commercial colorimetric kit according to the manufacturer’s instructions (#10720P, Human, Wiesbaden, Germany). Triglyceride concentration was then calculated as mg triglyceride per g liver tissue.

### 2.5. Histological Staining (Oil Red O, Sirius Red, PAS, Immunofluorescence)

For Oil Red O staining, frozen liver tissues were cut, fixed using 4% formaldehyde, stained with Oil Red O staining solution (0.36% Oil Red O in 60% isopropanol) for 60 min, destained with distilled water, and counterstained with hematoxylin.

For Sirius Red staining, formalin-fixed paraffin-embedded liver tissues were cut, stained with Sirius Red staining solution (0.1% Sirius Red in picric acid) for 45 min, and destained with 0.5% acetic acid for 1 min.

For Periodic acid-Schiff (PAS) staining, formalin-fixed paraffin-embedded colon tissues were cut, oxidized with 0.5% periodic acid for five minutes, stained with Schiff reagent for 15 min, destained with tap water, and counterstained with hematoxylin.

Immunofluorescence staining was performed using frozen tissue sections. Sections were fixed using 4% formaldehyde and incubated with primary antibody (Ly6G: 1:200, #551459, BD Biosciences, Franklin Lakes, NJ, USA; MUC2: 1:300, #ab76774, Abcam, Cambridge, UK; CLEC4F: 1:200, #AF2784, R&D Systems, Minneapolis, MN, USA) overnight at 4 °C. After washing, the sections were incubated with a fluorochrome-labeled secondary antibody (for Ly6G: anti-rat Cy3, 1:400; for MUC2: anti-rabbit Alexa Fluor 488; for Clec4f: anti-goat Alexa Fluor 546, 1:400; Thermo Fisher Scientific, Waltham, MA, USA) for 60 min at room temperature. Nuclei were counterstained using Vectashield Antifade Mounting Medium with DAPI (#H-1200-10, Vector Laboratories, Burlingame, CA, USA).

Imaging of histological sections was performed with an Axio Imager Z1 (Carl Zeiss, Oberkochen, Germany) for bright-field images or with an Axio Imager A2 (Carl Zeiss) for fluorescent images and processed using AxioVision LE64 version 4.9.1 software (Carl Zeiss). For quantification of stained histological sections (e.g., Ly6G-positive neutrophils, Clec4f-positive Kupffer cells), seven random pictures were taken for each mouse and the number of positively stained cells per view field counted. For each mouse, the mean value of positively stained cells per view field was plotted and depicted in the respective graphs.

### 2.6. Reverse Transcription-Quantitative PCR Analysis (RT-qPCR)

Total RNA was isolated using Invitrogen TRIzol Reagent (#15-596-018, Thermo Fisher Scientific). Reverse transcription was performed with random primers using Applied Biosystems High-Capacity cDNA Reverse Transcription Kit with RNase Inhibitor (#43-749-66, Thermo Fisher Scientific). Quantitative Real-Time PCR was performed using PowerUp SYBR Green Master Mix (#A25777, Thermo Fisher Scientific) in a QuantStudio 5 Real-Time PCR System (Thermo Fisher Scientific). Relative mRNA expression was calculated using the ΔΔCT method.

### 2.7. Flow Cytometry

First, capillary leukocytes were removed from the liver tissue by perfusion with PBS. Then, the liver tissue was digested at 37 °C for 45 min using Collagenase type 4 (#LS004189, Worthington, Lakewood, NJ, USA). Afterwards, the liver was minced through a 70 μm cell strainer and remaining erythrocytes were lysed using BD Pharm Lyse buffer (#555899, BD Biosciences). The resulting cell suspension was stained with fluorochrome-conjugated antibodies (1:300) for myeloid cells (“Mix1”) or lymphoid cells (“Mix2”) for 30 min at 4 °C.

“Mix1” contained MHCII FITC (#107605, BioLegend, San Diego, CA, USA), CD80 PE (#553769, BD Biosciences), Gr1.1 PerCP-Cy5.5 (#552093, BD Biosciences), F4/80 PE-Cy7 (#25-4801-82, Thermo Fisher Scientific), CD11c APC (#17-0114-82, Thermo Fisher Scientific), Ly6G Alexa Fluor 700 (#127622, BioLegend), CD45 APC-eFluor 780 (#47-0451-82, Thermo Fisher Scientific), CD11b V450 (#560456, BD Biosciences), and Calibrite APC beads (#340487, BD Biosciences).

“Mix 2” contained CD8a FITC (#11-0081-85, Thermo Fisher Scientific), CTLA-4 PE (#12-1522-83, Thermo Fisher Scientific), CD25 PerCP-Cy5.5 (#551071, BD Biosciences), NK1.1 PE-Cy7 (#25-5941-82, Thermo Fisher Scientific), CD3e APC (#12-0031-81, Thermo Fisher Scientific), CD19 Alexa Fluor 700 (#56-0193-80, Thermo Fisher Scientific), CD45 APC-eFluor 780 (#47-0451-82, Thermo Fisher Scientific), CD4 eFluor 450 (#48-0041-80, Thermo Fisher Scientific), TIM-3 BV650 (#747623, BD Biosciences), PD-1 PE-CF594 (#562523, BD Biosciences), and Calibrite APC beads (#340487, BD Biosciences).

Sample analysis was performed using a LSRFortessa Flow Cytometer (BD Biosciences) and FlowJo version 10.4.2 software (BD Biosciences). Cells were pre-gated on CD45 to identify leukocytes. Neutrophil granulocytes were determined as Ly6G^+^ cells. Ly6G^−^ cells were further gated on CD11b^high^ and F4/80^+^ to detect monocyte-derived macrophages. NK cells were identified as CD3^−^ NK 1.1^+^ cells, T cells as CD3^+^ NK1.1^−^ cells, and B cells as CD19^+^ CD3^−^ cells. Total cells per liver were calculated using Calibrite APC beads for calibration.

### 2.8. Isolation of Metagenomic DNA from Cecal Samples

Cecal samples were collected immediately after euthanizing mice and snap frozen. DNA was isolated using a modified version of a previously described protocol [20]. In brief, snap frozen samples were mixed with 600 µL stool DNA stabilizer, thawed, and transferred into tubes containing 500 mg silica/zirconia beads (0.1 mm diameter). Next, 250 μL 4 M guanidine thiocyanate in 0.1 M Tris (pH 7.5) and 500 μL 5% N-lauroyl sarcosine in 0.1 M PBS (pH 8.0) were added. Samples were incubated at 70 °C and 700 rpm for 60 min. A FastPrep instrument fitted with a 24 × 2 mL cooling adaptor filled with dry ice was used for cell disruption. The program was run three times for 40 s at 6.5 M/s. After each run, the cooling adapter was refilled with dry ice. An amount of 15 mg Polyvinylpyrrolidone was added and samples were vortexed, followed by 3 min centrifugation at 15,000× *g* and 4 °C. Approximately 650 µL of the supernatant were transferred into a new tube, which was centrifuged again for 3 min at 15,000× *g* and 4 °C. Subsequently, 500 µL of the supernatant was transferred into a new tube and 50 µg of RNase was added. After 20 min at 37 °C and 700 rpm, gDNA was isolated using the NucleoSpin gDNA Clean-up Kit (Macherey-Nagel, Düren, Germany). Isolation was performed according to the manufacturer’s protocol. DNA was eluted from columns twice, using 40 µL Elution buffer.

### 2.9. Illumina Sequencing of 16S rRNA Gene Amplicons

Library preparation and sequencing were performed as described in detail previously, using an automated platform (Biomek4000, Beckman Coulter) [21]. Briefly, the V3-V4 region of 16S rRNA genes was amplified in duplicates (15 + 10 cycles) following a two-step protocol using primers 341F-785R [22,23]. After purification using the AMPure XP system, sequencing was carried out with pooled samples in paired-end modus (PE300) using an Illumina MiSeq system according to the manufacturer’s instructions and 25% (*v*/*v*) PhiX standard library.

### 2.10. Sequencing Data Analysis

Raw reads were processed using an in-house developed pipeline (www.imngs.org, last accessed on 1 September 2021) as described previously [24], which is based on the UPARSE approach [25]. This pipeline was adapted to process 16S rRNA gene amplicon datasets with the following parameters: barcode mismatches = 1, abundance = 0.0025, 5-end trim = 0, 3-end trim = 0, trim score = 10, expected errors = 2, min. read length = 298, max. read length = 466, alignment min. %id = 70, min. ASV size = 4, OTU = yes, denoised amplicons = yes. The taxonomic annotation was performed using the Silva database. Following the IMNGS pipeline, the preprocessing was performed using Biom (version 2.1.10) [26]. R (version 4.0.3) was used to perform the downstream statistical analyses utilizing the packages Phyloseq (version 1.34.0) [27], microbiome (version 1.12.0), and ggplot2 (version 3.3.5). A parametric T test together with Benjamini-Hochberg *p* value adjustment was adopted to perform statistical comparisons.

Beta-diversity ordination was generated using Bray-Curtis distances and permutational multivariate ANOVA (ADONIS) was computed with 999 permutations using Vegan package (version 2.5.7), for which an R2 > 0.1 (effect size, 10%) and Pr(>F) value < 0.05 were considered as significant.

To observe ecosystem state variability, two alpha-diversity measures were evaluated, i.e., observed number of OTUs and Shannon effective (to estimate diversity). Shannon effective and observed OTUs were calculated using the R pipeline Rhea [28], and statistical comparisons between selected groups were performed using one-way ANOVA and the Sidak multiple comparisons test.

Differentially abundant OTUs were identified using DESeq2 (version 1.30.1). OTUs with Benjamini-Hochberg adjusted *p* value < 0.05 and estimated log2 fold-change > 2 were considered as significantly differentially abundant [29]. Plotting was done using EnhancedVolcano (version 1.8.0). LDA (linear discriminant analysis) scores obtained from LEfSe were used to estimate the effect size of the corresponding differentially abundant specific bacteria (*p* value < 0.05 and LDA score > 3.0) (http://huttenhower.sph.harvard.edu/galaxy/root?tool_id=lefse_upload, last accessed on 20 October 2021). The reference sequences of differentially abundant OTUs (*p* < 0.05) were validated with the 16S sequence database of EzBioCloud (https://www.ezbiocloud.net/, last accessed on 13 November 2021) using only valid names, and the percentage of sequence similarity was recorded.

### 2.11. Statistical Analysis

Data were analyzed using Prism software version 9.0.2 (GraphPad, San Diego, CA, USA) and are depicted as mean values with error bars indicating the standard error of the mean (SEM). Normal distribution within each group was verified using a Shapiro-Wilk test. Comparisons between two groups were analyzed by a two-tailed unpaired *t* test (with Welch’s correction if there was significantly different variance according to the F test) or a Mann-Whitney test (if not normal-distributed). Comparisons between more than two groups were analyzed using a one-way ANOVA with a Tukey’s multiple comparisons test or a Kruskal-Wallis test with a Dunn’s multiple comparison test (if normal distribution could not be verified). Differences were considered significant when *p* values were below 0.05.

## 3. Results

### 3.1. Long-Term Chronic-Plus-Binge Mouse Model of ALD Displays Liver Injury, Steatosis, and Neutrophil Infiltration

**Figure 1:**
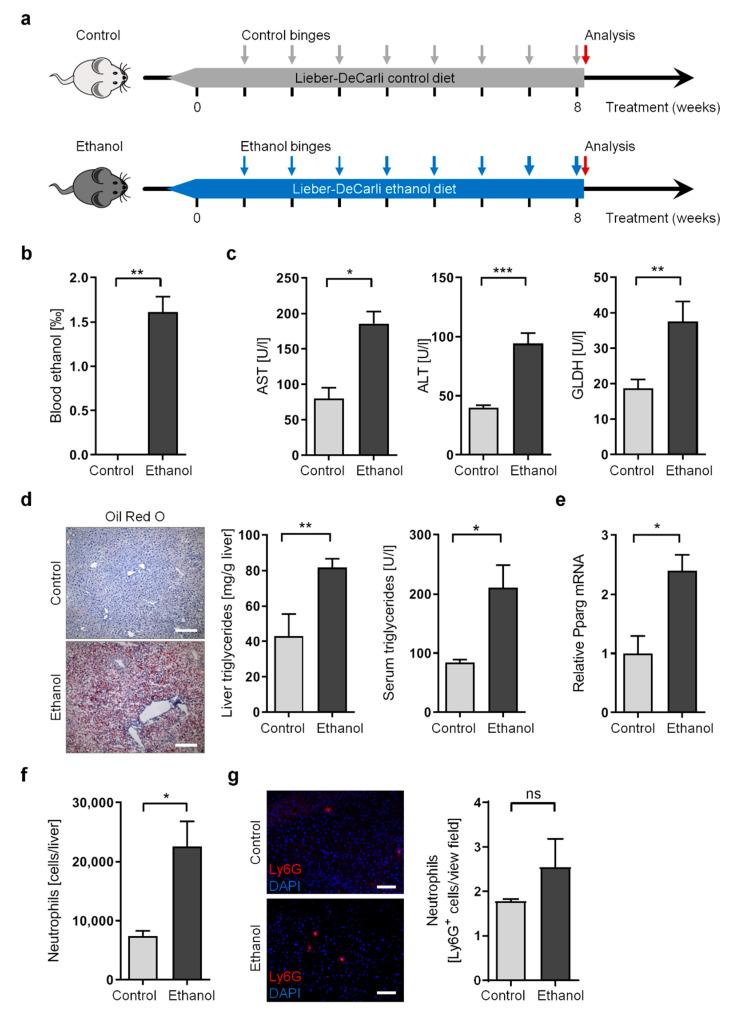
Long-term chronic-plus-binge alcohol feeding induces liver injury, steatosis, and neutrophil infiltration in mice. (**a**) Schematic overview of chronic-plus-binge alcohol feeding model. (**b**,**c**) Blood alcohol concentration (**b**) and serum concentration of AST, ALT, and GLDH (**c**) in ethanol-fed and control-fed WT mice. (**d**) Representative images of histological liver sections showing lipid droplets stained with Oil Red O (scale bars, 200 µm), as well as triglyceride concentration in livers and in serum from ethanol-fed and control-fed WT mice. (**e**) Relative mRNA expression of *Pparg* was determined by RT-qPCR analysis of liver tissue from ethanol-fed and control-fed WT mice and normalized to *B2m*. (**f**) Absolute numbers of neutrophil granulocytes (CD45^+^ Ly6G^+^ cells) per liver determined by flow cytometry in ethanol-fed and control-fed WT mice. (**g**) Representative images of histological liver sections stained for Ly6G (red) and nuclei (DAPI, blue), showing neutrophil granulocyte infiltration (scale bars, 100 µm) as well as quantification of neutrophil numbers per field of view (200× magnification) in livers from ethanol-fed and control-fed WT mice. Data represent mean ± SEM of at least five mice per group; *** *p* < 0.001, ** *p* < 0.01, * *p* < 0.05, ns = not significant.

To reproduce drinking patterns in human alcoholism, we utilized a novel long-term variation of the “NIAAA model” by combining 8-week ad libitum ethanol feeding (the Lieber-DeCarli ethanol diet) with multiple ethanol binges (once per week, eight binges in total) (Figure 1a). We believe this chronic-plus-binge model not only recapitulates drinking patterns in humans with chronic alcohol abuse, who are predisposed to develop alcoholic liver disease, but also aids in understanding long-term changes of the intestinal microbiome and the gut-liver axis, which in turn may impact disease progression in the liver.

To investigate the impact of this long-term chronic-plus-binge alcohol model on liver function, we compared ethanol-treated wild-type (WT) mice with control-treated WT mice, which underwent chronic liquid diet feeding (the Lieber-DeCarli control diet without ethanol) and multiple binge administrations lacking ethanol (Figure 1a). Mice were analyzed five hours after the last binge. Analysis of serum revealed strongly elevated blood alcohol concentration (BAC) of approximately 1.6% (Figure 1b). Concomitantly, analysis of liver enzymes in serum confirmed that chronic ethanol treatment triggered mild but consistent liver injury, as indicated by increased concentration of aspartate transaminase (AST), alanine transaminase (ALT), and glutamate dehydrogenase (GLDH) (Figure 1c).

We then evaluated the development of liver steatosis upon chronic alcohol treatment. As expected, ethanol-treated mice display enhanced accumulation of triglycerides and lipid droplets (macrosteatosis and microsteatosis) in the liver, as well as elevated serum triglycerides (Figure 1d). This was accompanied by increased expression of peroxisome proliferator activated receptor *gamma* (*Pparg*) in the liver (Figure 1e). While the main function of the transcription factor PPARG occurs in adipose tissue, PPARG has also been described as contributing to de novo lipogenesis and free fatty acid uptake in hepatocytes [30].

In addition to liver injury and steatosis, human ALD is characterized by progression towards alcoholic hepatitis with enhanced infiltration of neutrophil granulocytes. Indeed, we observed enhanced liver infiltration of neutrophil granulocytes in ethanol-treated mice, using either flow cytometry (Figure 1f) or immunofluorescence staining (Figure 1g). We also investigated whether disease progression in our long-term chronic-plus-binge model involved the occurrence of liver fibrosis. We observed significantly increased expression of Collagen I *alpha* 1 (*Col1a1*) in the livers of ethanol-treated mice (Figure S1a). However, enhanced fibrogenesis using Sirius Red staining for collagen fibers was barely detectable (Figure S1b).

These results indicate that this long-term chronic-plus-binge model recapitulates not only the drinking behavior of chronic alcoholism in humans, but also displays most of the molecular characteristics associated with the development of ALD in these patients, including elevated liver injury, steatosis, and neutrophil infiltration.

### 3.2. Chronic Alcohol Consumption and Nlrp6 Deficiency Modulate Intestinal Epithelium

To further investigate the impact of long-term alcohol abuse on ALD and to understand how this may be influenced by the gut-liver axis, we examined alterations in the intestinal epithelium. Since the NLRP6 inflammasome has been proposed to modulate intestinal homeostasis [31,32,33,34], we utilized *Nlrp6*-deficient mice (*Nlrp6*^−/−^) to determine the specific role of the NLRP6 inflammasome during chronic alcohol consumption.

**Figure 2:**
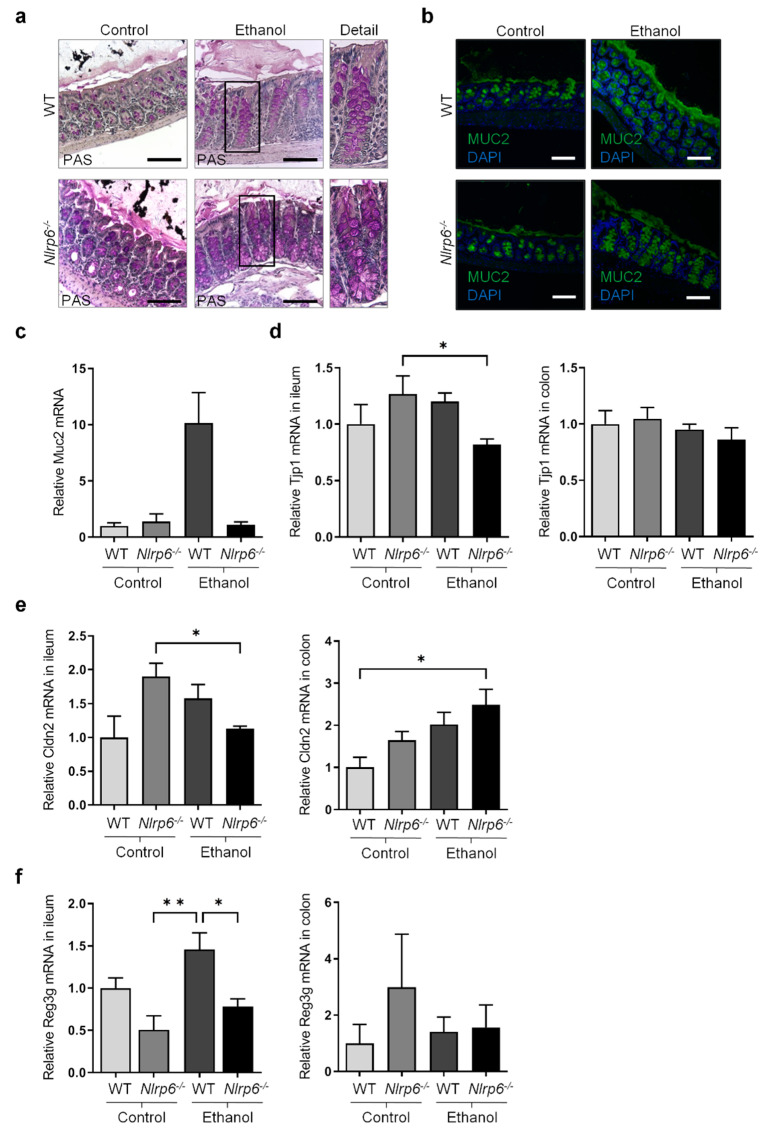
Chronic alcohol consumption and *Nlrp6* deficiency modulate intestinal epithelium in mice. (**a**,**b**) Representative images of histological colon sections stained with periodic acid-Schiff (PAS) (**a**) or stained for MUC2 (green) and nuclei (DAPI, blue) (**b**) from ethanol-fed and control-fed WT and *Nlrp6*^−/−^ deficient mice (scale bars, 100 µm, with area indicated by rectangle frame shown enlarged below “Detail”). (**c**–**f**) Relative mRNA expression of *Muc2* in ileum (**c**), *Tjp1* in ileum and colon (**d**), *Cldn2* in ileum and colon (**e**), and *Reg3g* in ileum and colon (**f**) was determined by RT-qPCR analysis of tissues from ethanol-fed and control-fed WT and *Nlrp6*^−/−^ mice and normalized to *B2m*. Data represent mean ± SEM of at least five mice per group; ** *p* < 0.01, * *p* < 0.05.

Using Periodic acid-Schiff (PAS) staining, we observed hyperplasia of goblet cells in the colon epithelium of *Nlrp6*-deficient mice compared to WT mice, irrespective of alcohol feeding (Figure 2a). Furthermore, using immunofluorescence staining for the mucus protein Mucin-2 (MUC2) in the colon, we detected a thickened mucus layer in WT mice upon chronic alcohol feeding (Figure 2b). This increased mucus layer was less pronounced in alcohol-fed *Nlrp6*-deficient mice. In line with this, we detected a trend towards increased expression of *Muc2* in the ileum of alcohol-fed WT mice compared to control-fed WT mice (Figure 2c). Notably, alcohol-fed *Nlrp6*-deficient mice showed a trend towards reduced *Muc2* expression compared to alcohol-fed WT mice (Figure 2c), consistent with a slightly reduced mucus layer in the colon (Figure 2b).

Next, we investigated the impact of chronic alcohol treatment or *Nlrp6* deficiency on the intestinal epithelial barrier. To this end, we evaluated the mRNA expression of the zonula occludens protein ZO-1 (*Tjp1*), a key component of tight junctions between enterocytes [6]. While *Tjp1* expression was unaltered in the colon, alcohol-fed *Nlrp6*-deficient mice showed reduced *Tjp1* expression in the ileum compared to control-fed *Nlrp6*^−/−^ animals (Figure 2d). Furthermore, mRNA level of Claudin-2 (*Cldn2*), a cation-channel-forming tight junction protein, was increased upon alcohol treatment and *Nlrp6* deficiency compared to control-fed WT mice in the colon (Figure 2e). In the ileum, alcohol-fed *Nlrp6*-deficient mice were found to express *Cldn2* at levels of control-fed WT mice (Figure 2e). Both reduced *Tjp1* and increased *Cldn2* expression in the ileum and the colon, respectively, constitute a first hint that the intestinal barrier function upon chronic alcohol exposure and *Nlrp6* deficiency might be compromised. However, additional future experiments are needed to draw a clear conclusion regarding the gut barrier.

Furthermore, reduced expression of bactericidal lectins (e.g., regenerating islet-derived 3 *beta*, *Reg3b*, and *gamma*, *Reg3g*) has been shown to be associated with bacterial translocation [4]. Interestingly, while we observed only a trend towards increased *Reg3g* mRNA expression upon chronic alcohol treatment in WT mice, *Nlrp6* deficiency caused a significant decrease of *Reg3g* expression in the ileum of alcohol-fed mice compared to corresponding WT mice (Figure 2f). Although not statistically significant, the expression of *Reg3b* followed a similar pattern in the ileum (Figure S2a).

These results suggest that some functions of the intestinal epithelium, such as mucus production, tight junction gene expression, and bactericidal lectin production, are partially compromised upon chronic alcohol feeding or in response to *Nlrp6* deletion. Potentially, this could negatively impact intestinal homeostasis and intestinal barrier function, possibly leading to enhanced bacterial translocation.

### 3.3. Chronic Alcohol Treatment and Nlrp6 Deletion Alter the Gut Microbiota

**Figure 3:**
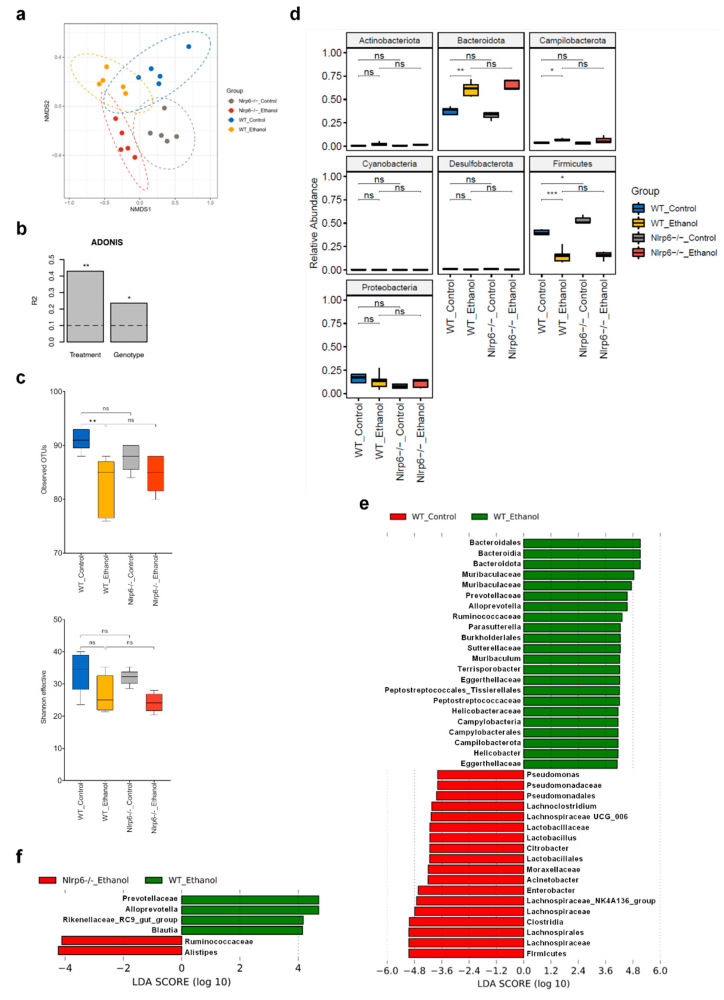
Cecal microbiota is altered markedly upon chronic alcohol treatment but only marginally by additional *Nlrp6* deletion. (**a**) NMDS analysis based on the Bray-Curtis distance metric from ethanol- and control-fed WT and *Nlrp6*^−/−^ mice. Every point represents one mouse, and ellipses indicate 95% confidence levels for each group of mice. (**b**) Permutational multivariate analysis of variance (ADONIS) using the Bray-Curtis metric of samples as in (**a**), considering factors treatment and genotype. (**c**) *Alpha*-diversity analysis of samples as in (**a**), based on the number of observed OTUs and the Shannon effective index. (**d**) Relative abundance of intestinal bacteria at the phylum level using samples as in (**a**). (**e**) LEfSe analysis comparing cecal stool samples from ethanol-fed WT with control-fed WT mice. (**f**) LEfSe analysis comparing cecal stool samples from ethanol-fed *Nlrp6*^−/−^ with ethanol-fed WT mice. Data represent results from five mice per group; bar plots display mean; box plots indicate median ± interquartile range and maximum/minimum; *** *p* < 0.001, ** *p* < 0.01, * *p* < 0.05, ns = not significant.

The data presented above indicate that chronic alcohol feeding and *Nlrp6* deficiency affect intestinal homeostasis and may alter the gut microbiota. Of note, to allow development of a potentially altered stable microbial community, we separated WT and *Nlrp6*^−/−^ mouse lines for at least two generations instead of utilizing littermate offspring from an initial heterozygous *Nlrp6*^+/−^ breeding pair. We then analyzed cecal samples using high-throughput 16S rRNA gene amplicon sequencing (V3-V4 hypervariable regions; 18,614 +/− 8394 high-quality sequences per sample; mean +/− SD). Non-metric multidimensional scaling (NMDS) analysis based on Bray-Curtis dissimilarity displayed distinct clustering of all four groups of mice, although some overlap between the 95% confidence level ellipses was observed (Figure 3a). In fact, individual analysis of WT mice showed complete separation between ethanol-fed and control-fed mice (Figure S3a), whereas separate analysis of ethanol-treated mice did not show clear separation between WT and *Nlrp6*-deficient mice (Figure S3b). To further investigate the relative impact of long-term alcohol feeding and NLRP6 inflammasome deficiency on microbiota diversity, we performed permutational multivariate analysis of variance (ADONIS). This analysis revealed that about 40% of microbiota diversity could be explained by the factor “treatment”, whereas only about 20% of variability was based on the factor “genotype” (Figure 3b).

Subsequent analysis of *alpha*-diversity suggested reduced diversity in ethanol- vs. control-treated WT mice according to the observed number of Operational Taxonomic Units (OTUs) (Figure 3c), although Shannon effective counts, which incorporate both species richness and evenness, showed only a reduction in median values without statistical significance (Figure 3c). Deletion of *Nlrp6* has been previously reported to trigger intestinal dysbiosis [32]. In control-treated mice, we detected a reduced number of observed bacterial species, although this difference did not reach statistical significance (Figure 3c). Notably, none of the *alpha*-diversity measures revealed a significant difference or a trend between ethanol-treated *Nlrp6*^−/−^ and corresponding WT mice, suggesting no significant additional impact of NLRP6 inflammasome deficiency on intestinal microbiota richness and evenness during chronic and excessive alcohol intake (Figure 3c).

To specifically assess alterations in the taxonomic composition, we first compared relative abundances of bacteria at the phylum level. As shown in Figure 3d, we observed significantly increased occurrence of bacteria belonging to the phyla *Bacteroidota* and *Campilobacterota*, whereas members of the phylum *Firmicutes* were significantly decreased in ethanol-treated compared to control-treated WT mice. We also detected increased relative abundances of bacteria belonging to the phylum *Firmicutes* in control-treated mice upon *Nlrp6* deletion (Figure 3d). In contrast, we observed no significant differences in relative abundance of any phylum between ethanol-treated *Nlrp6*^−/−^ and corresponding WT mice (Figure 3d).

Next, we utilized linear discriminant analysis effect size (LEfSe) to identify differences in pairwise comparisons at several taxonomic levels. As expected from previous analysis, comparison between ethanol-treated and control-treated WT mice revealed a large number of significant differences in relative abundances (Figure 3e and Supplementary Figure S3c). For example, we observed particularly increased relative abundances of the bacterial family *Muribaculaceae*, the genera *Parasutterella* and *Helicobacter*, and the species *Muribaculum intestinale* and *Terrisporobacter petrolearius*, whereas the family *Lachnospiraceae*, the genus *Lactobacillus*, and the species *Enterobacter cancerogenus*, *Kineothrix alysoides* and *Acinetobacter pittii* were decreased (Figure 3e and Supplementary Figure S3d). In contrast, when we compared ethanol-treated *Nlrp6*^−/−^ and corresponding WT mice, only very few significant differences in relative abundances were identified (Figure 3f and Supplementary Figure S3e). In particular, we detected increased relative abundances of the species *Alistipes putredinis*, while the family *Prevotellaceae* and the species *Phocaeicola vulgatus* were decreased (Figure 3f and Supplementary Figure S3f).

These results indicate that long-term alcohol feeding substantially alters intestinal microbiota composition in mice as reported previously [5,6], whereas absence of NLRP6 inflammasome signaling causes few alterations in gut microbiota of alcohol-fed mice.

### 3.4. Abrogation of NLRP6 Inflammasome Signaling Diminishes Liver Immune Cell Infiltration during Chronic Alcohol Consumption

Previous reports indicated that ALD in humans and mice involves activation of the inflammasome complex [10,11,35]. However, despite one report suggesting a protective role of the NLRP3 inflammasome during chronic alcohol treatment in mice [35], the impact of inflammasome activation, in particular the impact of the NLRP6 inflammasome, on ALD progression during long-term chronic alcohol abuse is unknown. Therefore, we investigated the effect of *Nlrp6* deletion on several parameters of ALD progression in our chronic-plus-binge model of chronic alcoholism (see Figure 1a).

**Figure 4:**
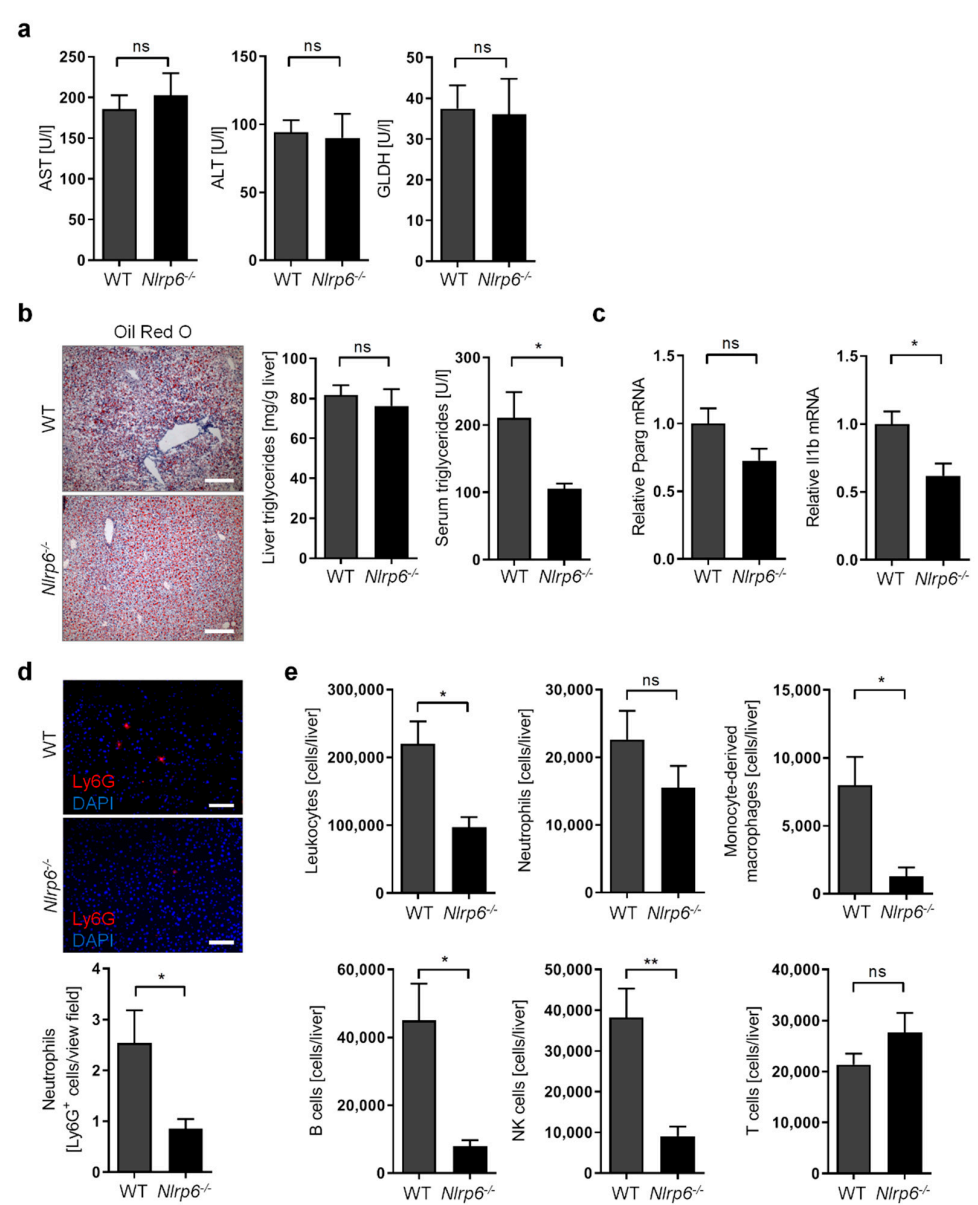
Abrogation of NLRP6 inflammasome signaling diminishes liver immune cell infiltration during chronic alcohol treatment in mice. (**a**) Serum concentration of AST, ALT, and GLDH in ethanol-fed WT and *Nlrp6*^−/−^ mice. (**b**) Representative images of histological liver sections showing lipid droplets stained with Oil Red O (scale bars, 200 µm), as well as triglyceride concentration in livers and in serum from ethanol-fed WT and *Nlrp6*^−/−^ mice. (**c**) Relative mRNA expression of *Pparg* and *Il1b* determined by RT-qPCR analysis of liver tissue from ethanol-fed WT and *Nlrp6*^−/−^ mice and normalized to *B2m*. (**d**) Representative images of histological liver sections stained for Ly6G (red) and nuclei (DAPI, blue) showing neutrophil granulocyte infiltration, as well as quantification of granulocyte numbers per view field (200× magnification) in livers from ethanol-fed WT and *Nlrp6*^−/−^ mice (scale bars, 100 µm). (**e**) Absolute numbers of leukocytes, neutrophil granulocytes, monocyte-derived macrophages, B cells, NK cells, and T cells per liver identified by flow cytometry in ethanol-fed WT and *Nlrp6*^−/−^ mice. Data represent mean ± SEM of at least five mice per group; ** *p* < 0.01, * *p* < 0.05, ns = not significant. Images and data for ethanol-fed WT mice are identical to those shown in Figure 1.

Surprisingly, we did not observe any impact of *Nlrp6* deficiency on liver injury as determined by serum concentration of AST, ALT, and GLDH (Figure 4a). Similarly, abrogation of NLRP6 inflammasome activity did not significantly alter the extent of liver steatosis and serum triglyceride concentration (Figure 4b). In line with this, the lipogenic transcription factor *Pparg* showed only a tendency but not a significant change towards decreased expression upon *Nlrp6* deletion in ethanol-fed mice (Figure 4c).

Next, we investigated the degree of liver inflammation as evidenced by the expression of several inflammation markers and the magnitude of immune cell infiltration into the liver. While the expression of *Il1b* was significantly reduced (Figure 4c), other well-established inflammation markers such as the tumor necrosis factor (*Tnf*, also known as TNF-*alpha*), interleukin 6 (*Il6*), and C-C motif chemokine ligand 2 (*Ccl2*) were not affected by the lack of NLRP6 signaling (Figure S4a). Furthermore, liver infiltration with neutrophil granulocytes was not significantly altered, but showed a trend towards reduced numbers in ethanol-fed mice upon *Nlrp6* deletion, as determined using immunofluorescence staining (Figure 4d) and flow cytometry (Figure 4e). We then explored whether liver infiltration with other immune cells was affected upon *Nlrp6* deficiency in ethanol-fed mice. Indeed, we observed significantly reduced overall numbers of liver-infiltrating leukocytes (Figure 4e). More specifically, the absence of NLRP6 led to reduced numbers of monocyte-derived macrophages, B lymphocytes, and natural killer cells (NK cells) in livers, whereas T lymphocyte remained unaffected (Figure 4e). Since liver-resident macrophages (i.e., Kupffer cells) can become depleted in response to significant liver injury, we also specifically determined Kupffer cell numbers in the livers of ethanol-fed mice. In line with a similar degree of liver injury (Figure 3a), we observed no alterations in Kupffer cell numbers using Clec4f as a specific Kupffer cell marker [36], as measured by immunofluorescence staining (Figure S4b) and RT-qPCR (Figure S4c).

These results suggest that the lack of NLRP6 inflammasome activity does not affect the degree of liver injury and steatosis during ALD, but slightly impairs inflammatory signaling and strongly reduces the extent of hepatic immune cell recruitment. Therefore, we conclude that NLRP6 inflammasome could play a disease-aggravating role during ALD by affecting the non-parenchymal liver environment.

## 4. Discussion

ALD is triggered by excessive and chronic alcohol consumption and can lead to severe medical conditions such as alcoholic hepatitis, liver fibrosis, liver cirrhosis, and even hepatocellular carcinoma. To better understand contributing pathways and to identify possible preventive or therapeutic interventions, the availability of suitable animal models is crucial.

In this report, we established a novel mouse model of chronic alcoholism that relies on long-term (8-week) alcohol consumption using the Lieber-DeCarli liquid diet complemented by multiple alcohol binge administration, similar to the previously published short-term (10-day) NIAAA model [16,18], thereby mimicking a pattern of chronic alcoholism in human patients with alcoholic hepatitis. Like the NIAAA model, we observed marked elevation of liver damage, extensive liver steatosis including macrosteatosis, and enhanced neutrophil infiltration. We also found significantly elevated collagen expression. However, this did not translate into significantly enhanced liver fibrogenesis. This finding seems to contrast with another published model of long-term (8-week) chronic-plus-binge alcohol administration [37]. 

Here, the authors reported the occurrence of mild fibrosis from using ethanol in either a single binge or multiple binges (twice per week). The lack in fibrogenesis cannot be explained, but differences in genetic background (C57BL/6N vs. C57BL/6J) and intestinal microbiome between animal facilities might account for these differences. However, this model recapitulates many features of human alcoholism and alcoholic steatohepatitis, and hence presents a suitable animal model for ALD.

While chronic alcohol abuse has many well-described direct effects on the liver, it also affects other organ systems, which may in turn impinge on liver function. The most important example is probably the intestine, which is affected by chronic alcohol consumption and has been recognized as playing a role in liver diseases, including ALD via a reciprocal impact, termed gut-liver axis [38]. We showed that alcohol treatment causes a thickening of the mucus layer and alterations in the expression of the tight junction protein Claudin-2. Surprisingly, we did not observe any alcohol-induced alterations in the expression of bactericidal lectins such as *Reg3b* or *Reg3g*, which has been reported previously [4]. These changes may indicate an altered intestinal epithelium function. However, it is unclear whether increased mucus production constitutes a direct effect of alcohol feeding or an indirect effect upon alterations of gut microbiota. For instance, increased relative abundance of *Akkermansia muciniphila* was reported to promote thickening of the mucus layer in a diet-induced model of obesity [39]. Although we did not detect this species, it is conceivable that specific microbiota alterations could trigger enhanced mucus production.

The gut microbiota not only plays a fundamental role for intestinal homeostasis but may also impinge on certain aspects of liver homeostasis during diseases that compromise intestinal barrier function. In particular, human patients with alcoholic liver disease as well as mice treated chronically with ethanol display evidence of impaired intestinal mucosa integrity and increased gut permeability leading to bacterial translocation [3,6]. Moreover, it was demonstrated that alcohol-induced liver inflammation in mice is mediated by altered intestinal microbiota [40]. Metagenomic analyses further uncovered that alcohol feeding reduces microbial diversity in mouse intestine [41]. In line with this, we observed reduced bacterial species richness in alcohol-treated mice, although the Shannon effective index showed only a trend towards reduced *alpha*-diversity. Furthermore, the occurrence of several bacterial phyla has been reported to be affected upon chronic alcohol consumption in humans and mice. For instance, several studies showed decreased relative abundances of bacteria belonging to the *Firmicutes* phylum [4,5]. Interestingly, microbiota analysis in our chronic-plus-binge alcohol model revealed reduced relative abundances of *Firmicutes* as the most significantly regulated phylum. Moreover, we detected an increased presence of *Bacteroidota*, consistent with an earlier study [4]. Since most intestinal bacteria in mice belong to the phyla *Bacteroidota* and *Firmicutes*, the ratio of these two phyla is of interest. In this regard, we observed a substantially decreased *Firmicutes*-to-*Bacteroidota* ratio, which was also detected by other groups [4,42]. Notably, *Helicobacter* and *Parasutterella* are among the bacterial genera that are more abundant upon chronic alcohol treatment and have been associated with inflammatory disease processes [43]. Hence, their increased presence may trigger elevated liver inflammation upon chronic alcohol exposure. In contrast, we observed a reduced presence of the bacterial family *Lachnospiraceae* and genus *Lactobacillus*, which belong to the *Firmicutes* phylum and were described as potentially beneficial during liver diseases [44,45].

Chronic liver diseases including ALD are characterized by an inflammatory phenotype, which involves immune cell translocation and activation, as well as by activation of inflammatory signaling pathways and secretion of inflammatory cytokines. One of the most important inflammatory pathways is the inflammasome, which mediates activation and secretion of inflammatory cytokines (IL1B and IL18) in response to activating signals such as bacterial LPS. While inflammasome complexes perform several beneficial functions during normal physiology in the liver and the gut, abnormally enhanced activity during inflammation-associated diseases can aggravate disease progression. Modulating inflammasome activity is, therefore, a potential therapeutic approach for interfering with detrimental inflammation in chronic liver diseases. Importantly, enhanced inflammasome activation was observed in human patients and mouse models for ALD and was shown to aggravate disease progression [10,11,35,46]. Notably, these results were either specific for the NLRP3 inflammasome or could not differentiate between different inflammasome complexes. In contrast, very little is known about the role of the NLRP6 inflammasome during alcohol-related chronic liver disease.

Previously, only a single study investigated the function of the NLRP6 inflammasome during ALD using the short-term NIAAA model and suggested a beneficial role during disease progression [13]. While the authors reported reduced liver damage, steatosis, and fibrosis upon NLRP6 overexpression [13], we did not observe any alteration in these disease characteristics upon abrogation of endogenous NLRP6 activity in our long-term chronic-plus-binge model of ALD. Moreover, this study suggested that enhancing NLRP6 activity alleviates detrimental liver inflammation in ALD, as evidenced by reduced neutrophil and macrophage infiltration, as well as reduced NF-κB activation in the liver [13]. In contrast, we clearly showed that a lack of NLRP6 signaling significantly reduces immune cell infiltration into livers, including neutrophils, macrophages, and natural killer cells. While the biological basis for this difference is not clear, our study used a well-described constitutive genetic deletion of endogenous mouse *Nlrp6* instead of non-physiological virus-mediated overexpression of an NLRP6 expression construct.

Furthermore, deficiency of NLRP6 inflammasome has previously been associated with intestinal dysbiosis [31,32]. Interestingly, more recent studies revealed that in littermate-controlled experiments, untreated WT and *Nlrp6*-deficient mice do not show significant differences in microbiota composition [47,48]. However, this appears to apply only under standardized specific pathogen-free (SPF) housing conditions that are devoid of pathobionts [49]. As our housing conditions are not completely devoid of pathobionts, and as we chose not to use littermate controls to allow development of an altered stable microbial community in the intestine, we expected a markedly altered gut microbiota upon *Nlrp6* deletion in our ALD model. In line with earlier reports, we observed intestinal hyperplasia and reduced mucus secretion in *Nlrp6*-deficient mice [31,33,34]. Surprisingly, although we detected some indication of increased intestinal permeability and reduced expression of antimicrobial peptides (e.g., *Reg3g*), we did not observe changes in intestinal microbial diversity upon Nlrp6 deficiency in our ALD model. Moreover, we detected only a few significant alterations in relative abundances of intestinal microbiota. In summary, during ALD, the lack of endogenous NLRP6 activity does not substantially modulate intestinal dysbiosis while protecting against extensive immune cell infiltration and hence disease progression in a presumably microbiota-independent manner.

One of the open questions of this study is the functional relevance of the observed alterations in the intestinal epithelium upon chronic alcohol treatment or *Nlrp6* deletion. Further investigation will be needed to clarify the impact on intestinal barrier function and bacterial translocation. Furthermore, while we observed an indication of reduced inflammatory signaling in *Nlrp6*-deficient mice during ALD, further transcriptomic approaches and protein-based analyses will be required to extend and validate this interesting finding. Importantly, although we detected severely reduced immune cell infiltration into livers upon *Nlrp6* deletion, it is unclear whether these *Nlrp6*-deficient immune cells may have altered or compromised functionality, as has been suggested previously [50]. Moreover, further analyses will be needed to clarify the role of the NLRP6 inflammasome in distinct cell types using either cell type-specific knockout models or functional analyses of individual cell populations ex vivo.

Although the role of NLRP6 inflammasome has not been well-studied in ALD, several reports have described its role during other types of chronic liver diseases such as non-alcoholic steatohepatitis (NASH). Using several mouse models for NASH, one study revealed that NLRP6 inflammasome activity performs beneficial functions in hepatocytes by reducing the extent of liver steatosis and inflammation [51]. While another study also claimed that NLRP6 activity negatively regulates NASH progression, the authors showed only enhanced liver damage upon constitutive *Nlrp6* deficiency and did not further investigate other parameters of disease progression such as steatosis, inflammation, or fibrosis [32]. Overall, NLRP6 seems to play a beneficial role during non-alcoholic chronic liver disease, which is probably due to a hepatocyte-specific function and modulation of hepatic lipid accumulation and inflammation.

In conclusion, depending on etiology, NLRP6 inflammasome activity may play detrimental or beneficial roles in chronic liver diseases. During chronic and excessive alcohol consumption, ubiquitous abrogation of *Nlrp6* in mice showed mild alterations of intestinal homeostasis but a clear reduction of hepatic immune cell recruitment, thereby suggesting a disease-aggravating role of the NLRP6 inflammasome during ALD.

## Data Availability

The high-throughput sequencing data of 16S rRNA gene amplicons were submitted to the EMBL-ENI European Nucleotide Archive (ENA) under accession number PRJEB48802.

## Supplementary Materials

The following supporting information can be downloaded at: https://www.mdpi.com/article/10.3390/cells11020182/s1, Figure S1: Long-term chronic-plus-binge alcohol feeding does not induce liver fibrosis in mice, Figure S2: Chronic alcohol consumption and *Nlrp6* deficiency modulate intestinal epithelium function in mice, Figure S3: Cecal microbiota is altered markedly upon chronic alcohol treatment but only marginally by additional *Nlrp6* deletion, Figure S4: Abrogation of NLRP6 inflammasome signaling does not affect inflammatory signaling and Kupffer cell numbers during chronic alcohol treatment in mice.
