# Supplementary material for: NLRP6 Inflammasome Modulates Disease Progression in a Chronic-Plus-Binge Mouse Model of Alcoholic Liver Disease
Source: Cells. 2022 Jan 6;11(2):182. doi: 10.3390/cells11020182 (PMC8773606; doi:10.3390/cells11020182)
Supplement: Supplementary file 1 [file cells-11-00182-s001.zip › cells-1498288-Supplementary Information.pdf]

Supplementary Materials

Supplementary figure legends

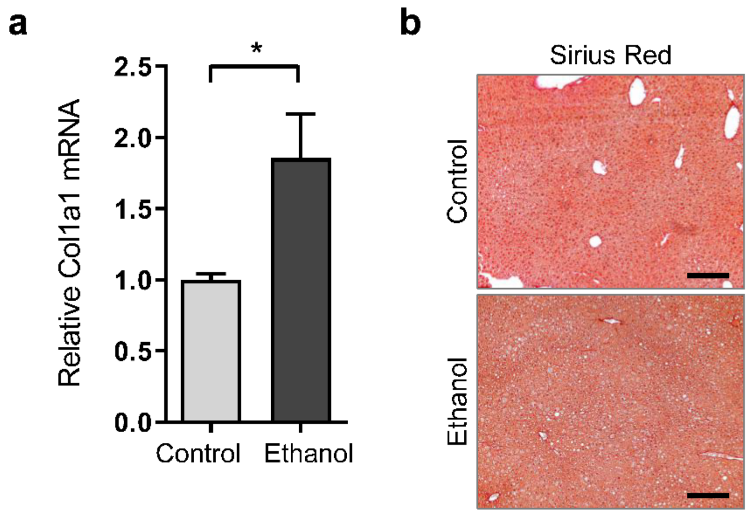

Figure S1: Long-term chronic-plus-binge alcohol feeding does not induce liver fibrosis in mice. (**a**) Relative mRNA expression of *Col1a1* was determined by RT-qPCR analysis of liver tissue from ethanol-fed and control-fed WT mice and normalized to *B2m*. (**b**) Representative images of histological liver sections stained with Sirius Red from ethanol-fed and control-fed WT mice (scale bars, 200 µm). Data represent mean ± SEM of at least five mice per group; * $p < 0.05$.

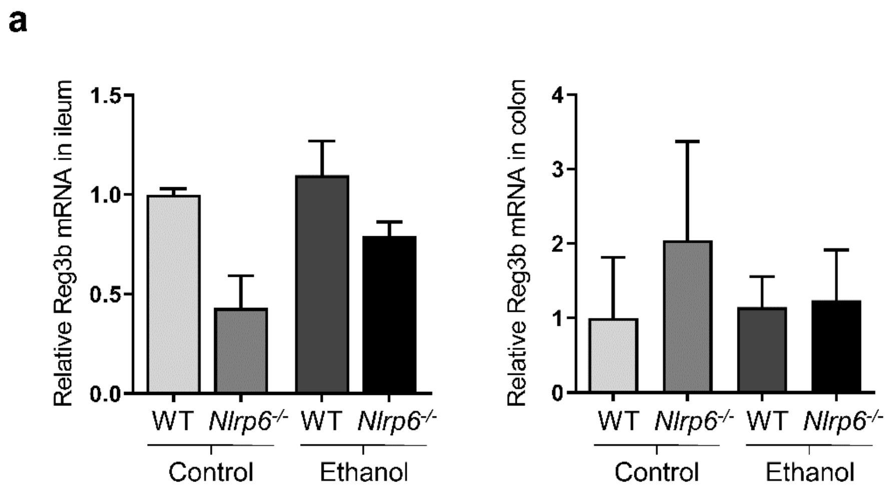

Figure S2: Chronic alcohol consumption and *Nlrp6* deficiency modulate intestinal epithelium function in mice. (**a**) Relative mRNA expression of *Reg3b* in ileum and colon was determined by RT-qPCR analysis of tissues from ethanol-fed and control-fed WT and *Nlrp6*$^{-/-}$ mice and normalized to *B2m*. Data represent mean ± SEM of at least five mice per group.

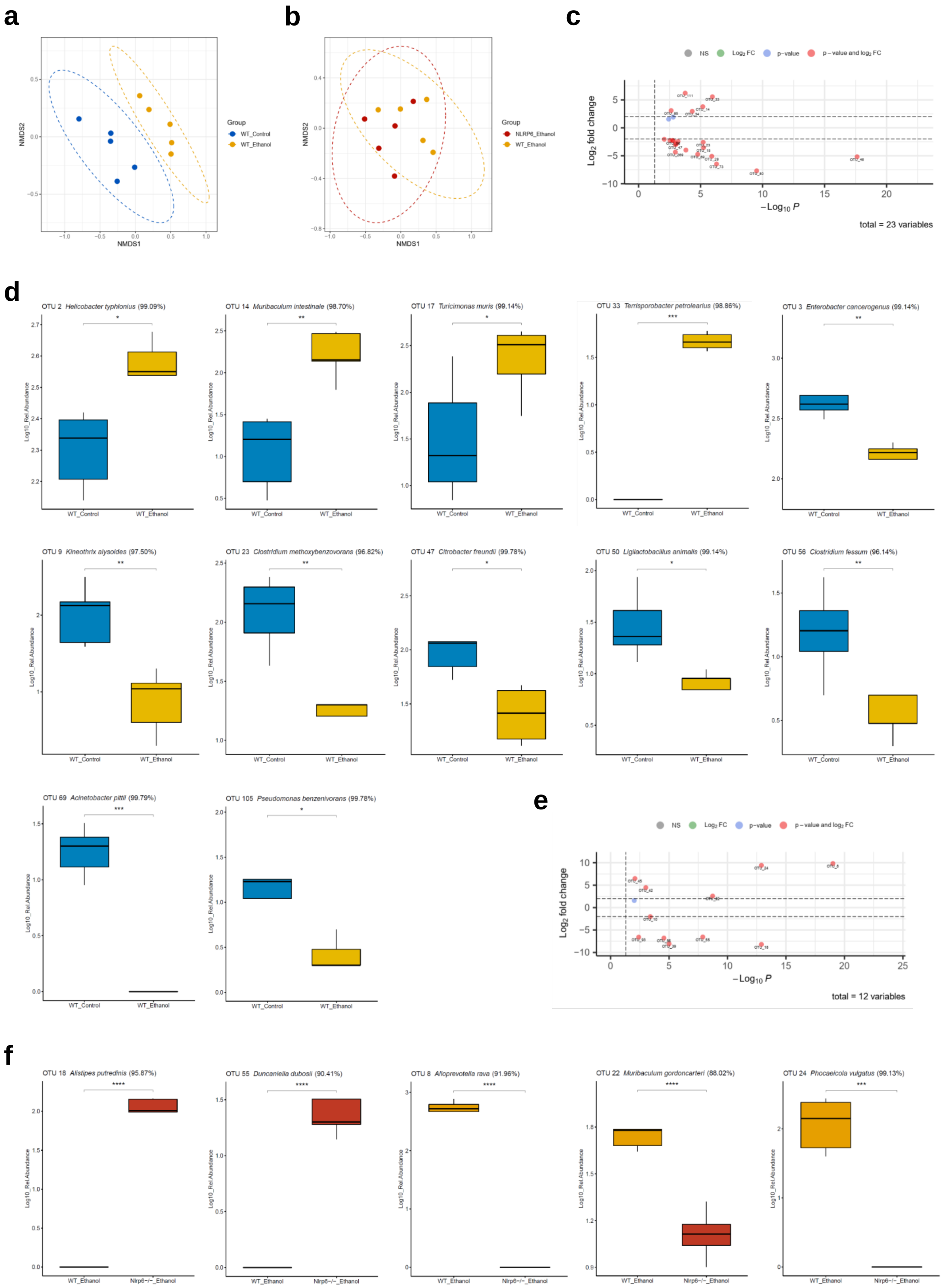

Figure S3: Cecal microbiota is altered markedly upon chronic alcohol treatment but only marginally by additional *Nlrp6* deletion. (**a, b**) NMDS analysis based on Bray-Curtis *beta*-diversity metric of cecal stool samples (**a**) from ethanol-fed WT and control-fed WT mice, or

(**b**) from ethanol-fed *Nlrp6*$^{-/-}$ and ethanol-fed WT mice. Every point represents microbiota composition from one mouse and ellipses indicate 95% confidence levels for each group of mice. (**c**) LEfSe analysis showing significantly altered OTUs comparing cecal stool samples from ethanol-fed WT with control-fed WT mice. (**d**) Relative abundance of significantly altered intestinal bacterial species comparing cecal stool samples from ethanol-fed WT with control-fed WT mice, based on LEfSe analysis and manually selected, with similarity of reference sequence for each OTU to 16S sequence database (EZBioCloud) in parentheses. (**e**) LEfSe analysis showing significantly altered OTUs comparing cecal stool samples from ethanol-fed *Nlrp6*$^{-/-}$ with ethanol-fed WT mice. (**f**) Relative abundance of significantly altered intestinal bacterial species as in (d), comparing cecal stool samples from ethanol-fed *Nlrp6*$^{-/-}$ with ethanol-fed WT mice. Data represent results from five mice per group; box plots indicate median ± interquartile range and maximum/minimum; **** $p < 0.0001$, *** $p < 0.001$, ** $p < 0.01$, * $p < 0.05$.

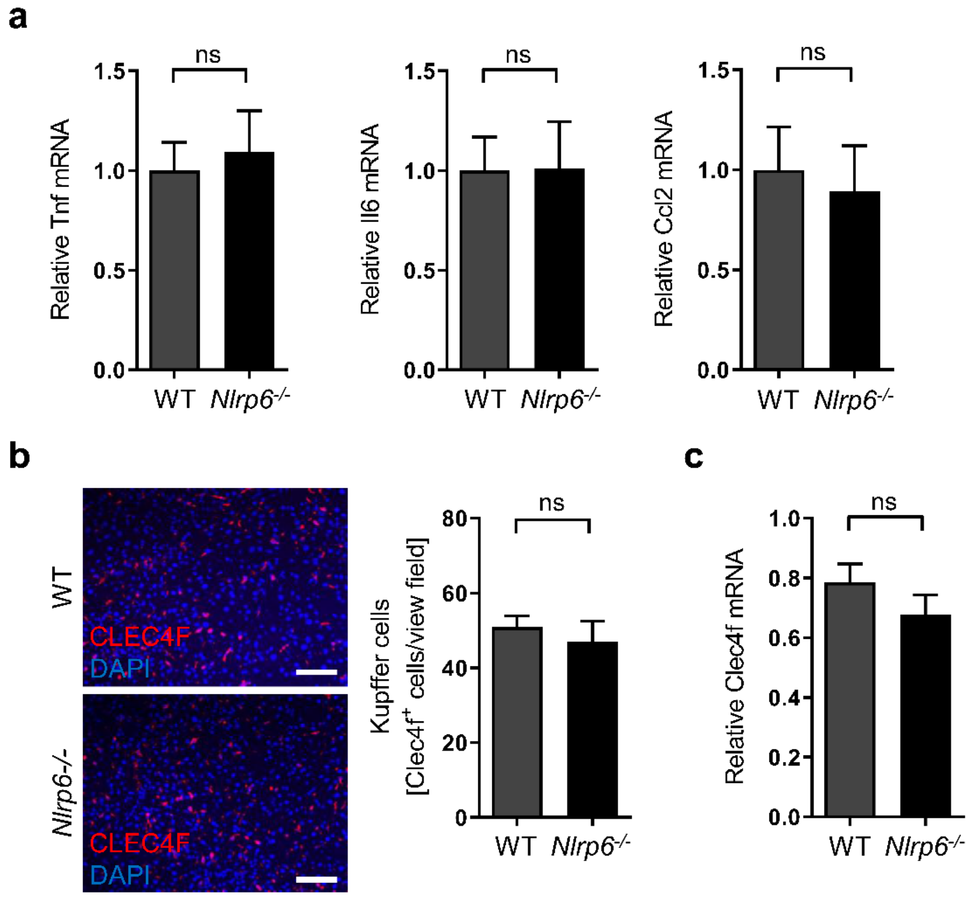

Figure S4: Abrogation of NLRP6 inflammasome signaling does not affect inflammatory signaling and Kupffer cell numbers during chronic alcohol treatment in mice. (**a**) Relative

mRNA expression of *Tnf*, *Il6*, and *Ccl2* was determined by RT-qPCR analysis of liver tissue from ethanol-fed WT and *Nlrp6*$^{-/-}$ mice and normalized to *B2m*. (**b**) Representative images of histological liver sections stained for CLEC4F (red) and nuclei (DAPI, blue) showing Kupffer cells (scale bars, 100 μm), as well as quantification of Kupffer cell numbers per field of view (200x magnification) in livers from ethanol-fed WT and *Nlrp6*-deficient mice. (**c**) Relative mRNA expression of *Clec4f* was determined by RT-qPCR analysis of liver tissue from ethanol-fed WT and *Nlrp6*-deficient mice and normalized to *B2m*. Data represent mean ± SEM of at least five mice per group; ns = not significant.
